# Supplementary material for: Association between oral microbiome and seven types of cancers in East Asian population: a two-sample Mendelian randomization analysis
Source: Front Mol Biosci. 2023 Nov 21;10:1327893. doi: 10.3389/fmolb.2023.1327893 (PMC10702768; doi:10.3389/fmolb.2023.1327893)
Supplement: Supplementary file 1 [file DataSheet1.ZIP › Supplementary Materials/MR plots of saliva/saliva═╝/Gastric cancer/pheno.3362_to_gastric cancer_leave_one_out.pdf]

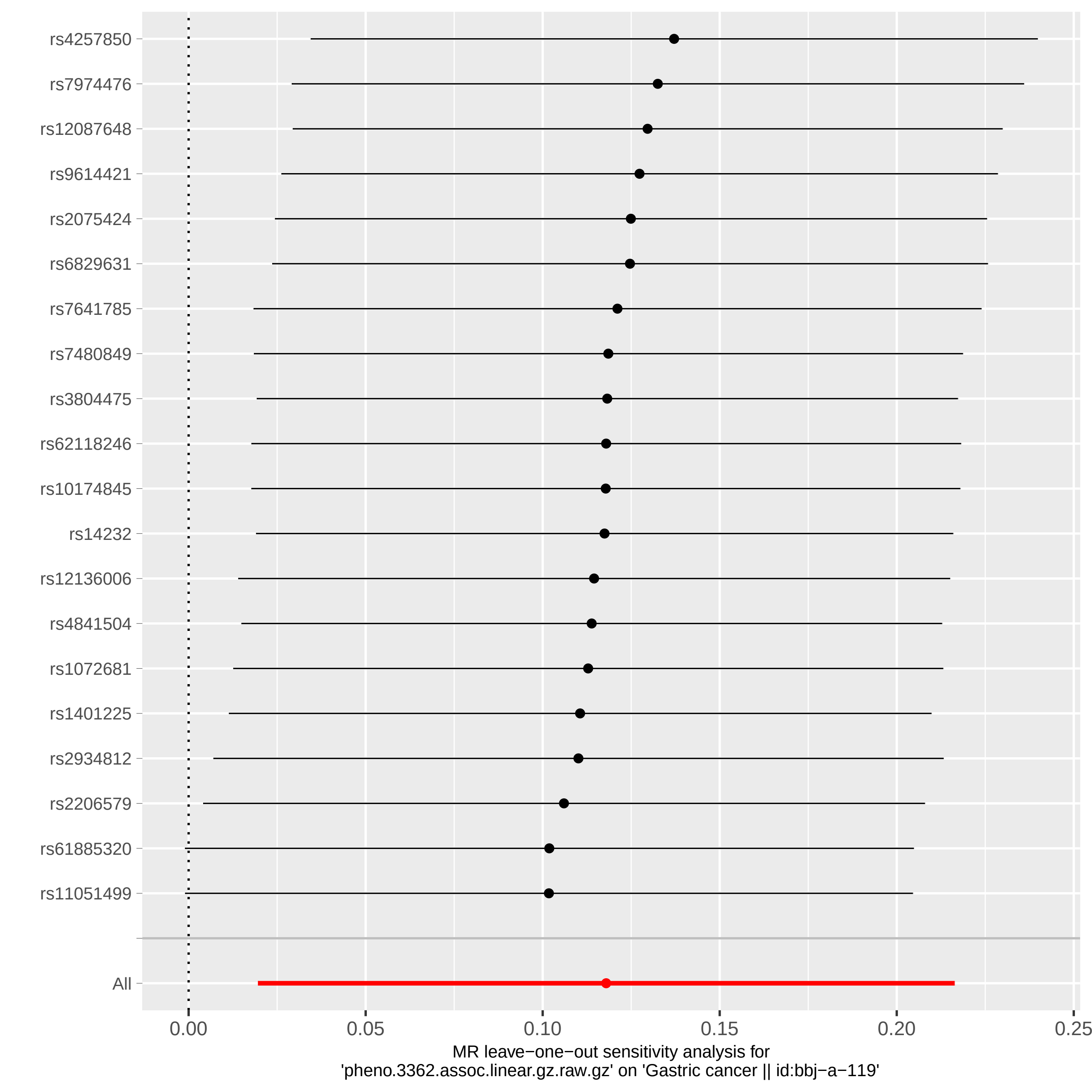
rs4257850
rs7974476
rs12087648
rs9614421
rs2075424
rs6829631
rs7641785
rs7480849
rs3804475
rs62118246
rs10174845
rs14232
rs12136006
rs4841504
rs1072681
rs1401225
rs2934812
rs2206579
rs61885320
rs11051499
All
0.00
0.05
0.10
0.15
0.20
0.25
MR leave−one−out sensitivity analysis for
'pheno.3362.assoc.linear.gz.raw.gz' on 'Gastric cancer || id:bbj−a−119'
